# Maternal depressive symptoms in and beyond the perinatal period: associations with infant and preschooler sleep.

**DOI:** 10.1093/sleep/zsae255

**Published:** 2024-10-29

**Authors:** Mikaela L Carter, Sarah-Jane Paine, Bronwyn M Sweeney, Joanne E Taylor, T Leigh Signal

**Affiliations:** Sleep/Wake Research Centre, Massey University, Wellington, New Zealand; Faculty of Medical and Health Sciences, University of Auckland, Auckland, New Zealand and; Sleep/Wake Research Centre, Massey University, Wellington, New Zealand; School of Psychology, Massey University, Palmerston North, New Zealand; Sleep/Wake Research Centre, Massey University, Wellington, New Zealand

**Keywords:** maternal mental health, depression, pregnancy, perinatal, child sleep, infants, preschoolers, sleep duration, sleep problems, health inequities

## Abstract

**Study Objectives:**

(1) To describe sleep in infancy and early childhood among children born to mothers with and without clinically significant depressive symptoms, and (2) to explore the relationships between maternal depressive symptoms and sleep patterns and problems during infancy and early childhood.

**Methods:**

Secondary analysis of longitudinal data from the Moe Kura: Mother and Child, Sleep and Wellbeing in Aotearoa/New Zealand study. Data were collected in pregnancy (T1), 12 weeks postpartum (T2), and 3 years post-birth (T3). Participants were 262 Māori and 594 non-Māori mother–child dyads. Chi-square and independent t-tests measured bivariate associations between maternal mood (T1, T2, and T3) and child sleep characteristics (T2 and T3). Binary logistic regression models examined longitudinal and concurrent associations between maternal depressive symptoms and infant and preschooler sleep. Adjusted models accounted for key socio-demographic variables, as well as infant sleep variables in preschooler models.

**Results:**

Bivariate associations were found between prior and concurrent depressive symptomology and many of the infant and preschooler sleep outcomes. In adjusted models, prenatal depressive symptoms remained independently associated with shorter-than-recommended sleep durations in preschoolers. In these models, concurrent depression was also associated with night waking, night LSRSP, and perceived sleep problems at 12 weeks postpartum, and CSHQ-determined and perceived sleep problems at 3 years post-birth.

**Conclusions:**

Longitudinal and cross-sectional associations were found between maternal depressive symptoms and child sleep. Sleep appears to be one pathway by which maternal depression confers risk for suboptimal child health outcomes. Findings support the need for earlier and better maternal mental health services.

## Graphical Abstract

**Graphical Abstract:**
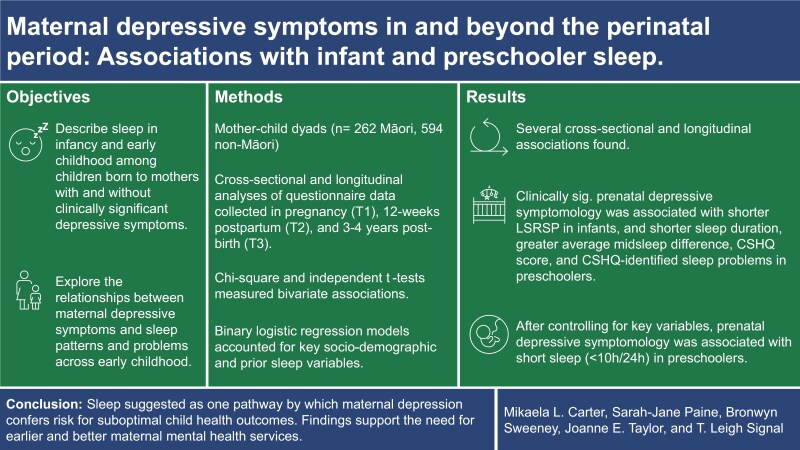

**Statement of Significance**

Maternal depression can have a deleterious effect on child well-being; child sleep is one potential mechanism by which risk is conferred. To our knowledge, this is the first study to explore longitudinal relationships between clinically significant prenatal depressive symptoms and child sleep in a large, socio-economically diverse sample with strong Indigenous representation. Findings indicate maternal depressive symptomology is associated with several child sleep outcomes in infancy and early childhood, including associations between prenatal depression and child sleep at 3 years. These findings highlight the need for population-based policies and interventions that support maternal mental health across and beyond the perinatal period.

Perinatal depression can have enduring deleterious effects that reverberate through family members and across time [1]. Studies suggest exposure to perinatal maternal depression can predispose children to later emotional, cognitive, and behavioral difficulties [2–5]. Identifying and understanding potential mechanisms of transmission can inform the type and timing of intervention required.

Sleep is one potential mechanism for which there is emerging evidence. Pediatric sleep health is integral to child development and sleep health is associated with a wide range of broader pediatric health outcomes [6]. The transactional model of infant sleep development [7] posits that infant sleep problems develop through a complex interplay of physiological and psychosocial factors, which include maternal depression. Postnatally, maternal depression is associated with lower personal, household, and social functioning [8], and may interfere with parent–infant interactions and caregiving activities, including sleep practices and routines [9]. However, there is evidence to suggest these processes begin in utero. Experimental animal studies indicate prenatal maternal distress is causally linked to sleep disturbance in offspring [10, 11]. One hypothesized mechanism, developed from animal studies, is that elevated exposure to glucocorticoids could disrupt development of the fetus’ hypothalamic–pituitary–adrenal axis and its diurnal pattern [12]. Though undoubtably more complex, there is some evidence to suggest a corresponding mechanism in humans [13].

Irrespective of the specific pathway, higher levels of maternal depression appear to confer risk for subsequent infant sleep problems. Bat-Pitault et al. [14] investigated the sleep architecture of newborn and six-month-old infants born to mothers diagnosed with major depressive disorder (MDD), compared to matched infants born to mothers with no history of depression (total *n *= 64). Polysomnography (PSG) comparisons showed group-based differences in sleep architecture at both time points. The infants of mothers with MDD diagnoses exhibited more disturbed sleep, lower total sleep time, higher wake time, and lower sleep efficiency than their clinically and demographically matched peers whose mothers had no depression history. These findings were in line with a similar study using actigraphy (*n* = 18) [15]. Though informative, these studies were limited in that infants in the “maternal depression” groups were not necessarily exposed to depression in utero, as inclusion criteria allowed for historic and possibly remitted MDD diagnoses.

Studies using objective sleep measures are also limited by small sample sizes. Parent-report measures of infant sleep have enabled larger-scale studies of these posited links. In a study of 312 mother-infant dyads, Dias and Figueiredo [16] reported maternal depressive symptoms during the third trimester of pregnancy predicted more unsettled sleep and more daytime sleepiness in infants at 3 and 6 months of age. Similarly, prenatal depression was associated with long sleep onset latency and irregular sleep routines in 3-month-old infants [17]. However, these studies found several other aspects of infant sleep, including night waking, showed no association with prenatal depression. Other studies fail to support the purported relationship entirely. Galbally et al. [18] found neither maternal prenatal depression nor prenatal antidepressant use predicted later infant sleep outcomes. Further research into the maternal depression–infant sleep relationship is needed.

In addition to adding valuable evidence to the limited and mixed findings in current studies, it is important for research to consider the potential impact of maternal depression on child sleep beyond infancy. Across the first 3 years of life, sleep undergoes rapid development. Sleep/wake patterns change markedly in this period, and exhibit great variability [19]. A small number of studies have looked at the relationship between maternal prenatal depressive symptoms and sleep in older children, including two prospective cohort studies [20, 21]. Toffol et al. [20] found children of women with clinically significant depressive symptomology during pregnancy had shorter nocturnal sleep duration, longer sleep onset latency, higher odds of more frequent night waking, and higher odds of having a sleep disorder at age 3.5 years, compared to children of women with low symptomology during pregnancy. The study controlled for several obstetric and psychosocial covariates, and concurrent maternal depression at 3.5 years, but not for depressive symptoms during the postnatal period. O’Connor et al. [21] looked at the impact of maternal prenatal anxiety and depression on child sleep at 18 months (*n* = 7458) and 30 months (*n* = 6829) after controlling for several obstetric and psychosocial covariates, as well as prenatal and postnatal depression, but not concurrent depression. Here, total sleep problems but not total sleep duration, nor night wakings were associated with maternal mental distress during pregnancy.

An important limitation characterizing most research in this field is the predominance of white mothers in moderate—high socioeconomic positions (SEPs) in study samples. Indeed, in many cases, ethnicity and/or SEP are not reported. In particular, Indigenous groups are not widely represented in this body of literature, which limits the ability of current findings to inform appropriate and effective prevention and intervention programs.

In sum, further research is needed to better elucidate the posited relationship between perinatal depression and child sleep over time, particularly research with extended timeframes and more diverse samples. The current study is guided by Kaupapa Māori research theory, an Indigenous research paradigm that is responsive to Māori needs and committed to providing outcomes that are useful to Māori, the Indigenous peoples of Aotearoa New Zealand (NZ) [22–24]. Māori represent approximately 30% of the current sample and the SEP distribution of the sample is broadly representative of Māori and non-Māori populations in NZ. Here, secondary analysis of the Moe Kura: Mother and Child, Sleep and Wellbeing in Aotearoa/New Zealand (*Moe Kura*) dataset was used to (1) report sleep characteristics during infancy and early childhood of children born to mothers with and without clinically significant depressive symptomology at three time points (third trimester of pregnancy, 12 weeks postnatal, and 3 years post-birth); and (2) explore maternal perinatal depressive symptomology as a predictor of sleep patterns and problems during infancy and early childhood, controlling for maternal depressive symptoms at subsequent time points.

## Methods

This research was part of *Moe Kura*, a longitudinal, observational, cohort study exploring the role of sleep in the health and well-being of Māori and non-Māori mothers and their children in NZ. The study followed mother–child dyads from pregnancy until the *Moe Kura* children were approximately 3 years old, with 4 survey-based data collection waves between October 2009 and April 2015 (see Signal et al. [24] for a detailed description of the recruitment process). Guided by Kaupapa Māori epidemiological methodology, three key principles underpin *Moe Kura*: (1) Māori participation and control at all levels and stages of the research; (2) appropriate collection of ethnicity data to identify and monitor health disparities; and (3) equal explanatory and analytical power for Māori and non-Māori. These principles and the broader methodological approach are explained more thoroughly elsewhere [24, 25]. The current research used data from 35 to 37 weeks’ gestation (T1), 12 weeks postnatal (T2), and 3 years post-birth (T3) and is covered by the study’s ethical approval (HDEC; CEN 09/09/070).

### Measures

#### Maternal and child demographics.

Participating mothers self-identified their own and their children’s ethnicity in a question taken from the NZ Census: “Which ethnic group do you [does your child] belong to? Mark the space or spaces that apply to you [your child].” Participants were categorized as Māori if they marked “Māori” alone or with (an)other ethnic group(s), and everyone else was categorized as non-Māori [26]. Maternal ethnicity was measured at T1 and child ethnicity was measured at T2.

SEP was measured at T1 with the New Zealand Index of Deprivation (NZDep2006). NZDep is a widely used area-based measure of socioeconomic deprivation in NZ; it uses nine census variables to estimate the level of material deprivation for people, based on home address, in each small area. Each area is assigned a decile from 1 (least deprived) to 10 (most deprived). Consistent with other research (Vaipuna et al. [27]), these deciles were collapsed into three levels of socioeconomic deprivation: low (deciles 1–3), medium (deciles 4–7), and high (deciles 8–10).

Other demographic measures were maternal age (years), parity at T1 (nulliparous or multiparous), whether the mother was caring for child(ren) younger than the *Moe Kura* child at T3 (yes/no), child age (gestational weeks at T1, weeks at T2, years at T3), child sex, and child gestational age at birth (weeks).

#### Maternal depressive symptoms.

Maternal depressive symptoms at T1 and T2 were measured with the Edinburgh Postnatal Depression Scale (EPDS) [28]. The EPDS is a widely used screening tool that assesses common symptoms of depression in the pre- and postnatal periods [29, 30]. The EPDS is comprised of 10 items scored on a 4-point Likert-type scale. Possible scores range from 0 to 30, with higher scores indicating more distress. Scores of 13 or more are considered clinically significant [31, 32]. At 3 years post-birth (T3), the EPDS was not considered an appropriate measure. Instead, depressive symptoms at T3 were measured with the Kessler 10 Item Scale (K-10) [33, 34]. A common measure of depressive symptoms, the K-10 can be used to assess symptomatology in the past month. The K-10 was developed for use in the annual US National Health Survey [34] and has been used in NZ national surveys since 2003/2004 [35]. Possible scores range from 0 to 40, with higher scores indicating more distress. Scores of 12 or more are considered clinically significant [35]. For the purposes of this study, some analyses are reported using mothers’ scores on the EPDS and K-10 (a continuous variable indicating the extent of depressive symptoms), whereas other analyses dichotomized mothers’ scores into categories based on whether clinically significant depressive symptomology was present (≥13 EPDS, ≥12 K-10), or absent (scores <13 EPDS, <12 K-10).

#### Child sleep.

A range of sleep variables were selected to reflect the multifaceted nature of pediatric sleep health and the complex physiological and psychosocial pathways by which depression may influence its development.

Infant sleep variables (T2) were night waking, longest continuous self-regulated sleep period (LSRSP) at night and during the day, and maternally perceived sleep problem. Each was assessed with a single multi-choice questionnaire item and subsequently dichotomized based on relationships seen with parent reports of problematic sleep [36], and National Sleep Foundation (NSF) guidelines [37]. *Night waking* was assessed with the question “how many times does your baby usually wake up between 10 pm and 6 am?” Response options were “0/not at all, 1, 2, 3, 4 or more times.” *Night waking* was dichotomized to 3+ vs. 0–2 wakings. LSRSPs were assessed with questions “what is the longest stretch of time that your baby is asleep during the night without waking up?” and “what is the longest stretch of time that your baby is asleep during the day without waking up?” Response options were “(Less than 30 minutes, 30 minutes–1 hour, 1–2 hours, 2–3 hours, 3–4 hours, more than 4 hours).” *Night LSRSP* was dichotomized to ≤4 vs. >4 hours, *Day LSRSP* was dichotomized to ≤1 hour vs. >1 hour. *Perceived sleep problem* was assessed with a single item from the Brief Infant Sleep Questionnaire [38]: “In general, do you consider your child’s sleep a problem?” Response options were “a very serious problem, a small problem, not a problem at all”; affirmative responses were combined to create the binary variable.

Preschooler sleep variables at T3 pertained to sleep duration and timing, as well as clinically significant and maternally perceived sleep problems. Most variables were obtained from the Children’s Sleep Habits Questionnaire (CSHQ); a widely used parent-report measure designed to screen for pediatric sleep problems [39]. Originally designed for use with school-aged children, the CSHQ has been validated for use with toddlers and preschoolers [40]. It is comprised of 45 items examining pediatric sleep behaviors occurring in a “typical” recent week. Most items are rated on a three-point scale: usually (5–7 times/week); sometimes (2–4 times/week); and rarely (0–1 time/week). Open-response items assess sleep timing and duration. Thirty-three CSHQ items were summed to produce a total CSHQ score, with scores able to range from 33 to 99. A higher score is indicative of more disturbed sleep, and a total CSHQ score of over 41 is thought to indicate a clinically significant pediatric sleep problem [39, 41].

Week and weekend *sleep durations* were assessed with CSHQ items pertaining to usual sleep durations per 24 hours, including naps. Consistent with NSF sleep duration guidelines [37] and prior research [42], *sleep duration* variables were categorized as short (<10 hours)/recommended (10–13 hours)/long (>13 hours). *Midsleep difference* and *social jetlag* were calculated from CSHQ items on wake times during the week (Monday to Friday) and on weekends (Saturday and Sunday), and additional items assessing sleep start times (as opposed to bedtimes) on week (Sunday to Thursday) and weekend evenings (Friday and Saturday). *Midsleep difference* was calculated as the absolute difference, in hours, between weeknight and weekend midsleep times (i.e. the midpoint between a child’s usual sleep start and wake times). Midsleep difference was dichotomized to produce a *social jetlag* (≥1 vs. <1 hour) variable (Muller et al. [43]). *CSHQ-indicated sleep problem* was defined as CSHQ score > 41 [39,41]. *Perceived sleep problem* was assessed with an additional item asking how much of a problem respondents found their child’s sleeping patterns or habits; “no problem, small problem, moderate problem, large problem.” Responses were dichotomized to moderate/large vs. no/small problem.

### Statistical analyses

All analyses were conducted using IBM SPSS statistical software (Version 28.0). Descriptive statistics are presented by maternal ethnicity (Māori/non-Māori, as measured at T1). Bivariate associations between maternal mood (clinically significant depressive symptoms: Yes/No) and sleep characteristics at each time point are then reported. Independent t-tests were used for continuous demographic variables, and Pearson chi-square tests for categorical demographic variables. Finally, binary logistic regression models were fitted to produce adjusted odds ratios and 95% CI for all binary sleep outcome variables. Independent variables in *infant models* were pre- and postnatal EPDS scores (continuous variables), maternal ethnicity at T1, maternal age at T1, NZDep level at T1, child age at T2, and child sex. *Preschooler models* included the same independent variables, plus K-10 scores (continuous variable) and infant (T2) sleep variables (night waking, night LSRSP, day LSRSP and perceived sleep problem). Child age in preschooler models was measured at T3. To ensure adequate events per variable (EPV), some demographic variables (including parity, gestational age at birth, and birth weight) were not included. Short sleep models compared short (<10 hours) to recommended (10–13 hours), excluding long (>13 hours) sleep durations per 24 hours. Long sleep was excluded from all analyses due to low rates/insufficient power (total *n* = 44 weeks, *n* = 39 weekends). Social jetlag (*n* = 51 ≥1 hour) was not modeled due to insufficient EPV. We conducted linear regression diagnostics to obtain tolerance statistics for the independent variables used in infant and preschooler models. All tolerance statistics were above the threshold of .20, indicating no significant multicollinearity [44, 45].

## Results

Participants were 856 women (*n* = 262 Māori and *n* = 594 non-Māori) who completed questionnaires at each of the three data collection points. Prior *Moe Kura* research showed that this dataset represents 75% of the baseline cohort [46]. A comparison of T1 characteristics between those with complete and incomplete datasets [46] showed that participants who could not be included in analyses were more likely to be younger (*p* < .001), identify as Māori (*p* < .001), live in a more deprived area (*p* < .001), and have a higher prenatal EPDS score (*p* = .030). There were no attrition-related differences in parity, or prevalence of clinically significant depressive symptoms (EPDS score ≥ 13) in pregnancy.

### Sample characteristics

The demographic characteristics of the Māori and non-Māori samples at each data collection point are presented in Table 1. Māori mothers were younger on average and more likely to be living in high-deprivation areas. Māori mothers reported more depressive symptoms during pregnancy (T1) and at 3 years post-birth (T3), but not at 12 weeks postnatal (T2). Non-Māori mothers were more likely to be nulliparous at T1 (*p* = .019). There was no difference in the likelihood of having child(ren) younger than the *Moe Kura* child at T3 by ethnicity. The children of Māori and non-Māori mothers did not differ in sex, age, or gestational age at delivery.

**Table 1.** Sample characteristics of mothers and children by maternal ethnicity

	T1 (pregnancy)				T2 (~12 weeks postpartum)				T3 (child age ~3 years)			
	Mean ± SD or *n* %				Mean ± SD or *n* (%)				Mean ± SD or *n* (%)			
	Māori	Non-Māori	Total	*p* value	Māori^a^	Non-Māori^a^	Total	*p* value	Māori^a^	Non-Māori^a^	Total	*p* value
	*n* = 262	*n* = 594	*n* = 856		*n* = 262	*n* = 594	*n* = 856		*n* = 262	*n* = 594	*n* = 856	
Maternal characteristics												
Age (years)	28.45 ± 6.25	31.91 ± 5.06	30.85 ± 5.68	**<.001**	28.75 (6.16)	32.22 (5.07)	31.16 (5.66)	**<.001**	31.60 ± 6.29	35.15 ± 5.07	34.07 ± 5.71	**<.001**
NZDep^b^				**<.001**				**<.001**				**<.001**
Low deprivation (deciles 1–3)	44 (16.8)	241 (40.6)	285 (33.3)		43 (16.4)	240 (40.5)	283 (33.1)		44 (17.4)	279 (49.0)	323 (39.3)	
Moderate (deciles 4–7)	104 (39.7)	253 (42.7)	357 (41.7)		105 (40.1)	252 (42.5)	357 (41.8)		93 (36.8)	207 (36.4)	300 (35.0)	
High deprivation (deciles 8–10)	114 (43.5)	99 (16.7)	213 (24.9)		114 (43.5)	101 (17.0)	215 (25.1)		116 (45.8)	83 (14.6)	199 (23.2)	
Parity^c^												
Nulliparous at T1	113 (44.8)	316 (53.7)	429 (50.1)	**.019**								
Younger child(ren) at T3									90 (34.6)	323 (39.2)	322 (37.6)	.205
Psychological distress^d^												
EPDS/K-10 mean	8.80 ± 5.02	7.61 ± 4.71	7.97 ± 4.83	**<.001**	6.09 ± 4.78	5.46 ± 4.01	5.65 ± 4.27	.064	7.57 ± 6.16	6.12 ± 4.97	6.56 ± 5.40	**<.001**
EPDS/K-10 clinically significant^e^	57 (22.0)	86 (14.5)	143 (16.8)	**.007**	31 (11.9)	40 (6.8)	71 (8.3)	**.013**	57 (21.8)	77 (13.0)	134 (15.7)	**<.001**
Child characteristics												
Sex (f)^f^									127 (48.8)	302 (51.5)	429 (50.7)	.407
Ethnicity												
Māori					248 (94.7)	52 (8.8)	300 (35.0)	**<.001**	253 (96.9)	58 (9.8)	311 (36.4)	**<.001**
Non-Māori					14 (5.3)	542 (91.2)	556 (65.0)		8 (3.1)	535 (90.2)	543 (63.6)	
Gestational or child age^g^	35.91 ± 0.97	35.82 ± 0.85	35.85 ± 0.89	.177	12.05 ± 1.28	12.05 ± 1.18	12.05 ± 1.21	.963	3.13 ± 0.31	3.15 ± 0.27	3.15 ± 0.28	.326
Gestational age at delivery (weeks)^h^					39.79 ± 1.42	39.69 ± 1.52	39.71 ± 1.49	.372				

Data presented as mean (SD) except where indicated as *n* (%). Bold values indicated statistical significance.

^a^Maternal ethnicity as reported during pregnancy.

^b^1, 1, and 34 missing cases for NZDep categories at T1, T2, and T3, respectively.

^c^15 and 4 missing cases for parity status at T1 and T3, respectively.

^d^3 and 3 missing cases for psychological distress scores at T1 and T2, respectively.

^e^Defined as EPDS ≥ 13, K-10 ≥ 12.

^f^Child sex was first measured at T3, 10 missing.

^g^Gestational age at T1 and child age at T2 measured in weeks, child age at T3 measured in years.

^h^5 missing cases for gestational age at delivery.

### Bivariate associations

The sleep characteristics of infants and preschoolers by maternal mental health status (i.e. with or without clinically significant depressive symptoms as indicated by the EPDS/K-10) are presented in Table 2. *Prenatal depression:* children born to mothers who had clinically significant depressive symptoms in pregnancy (T1) were more likely to have shorter (≤4 hours) night LSRSP at 12 weeks (T2). At 3 years (T3), their average weekday and weekend sleep durations were shorter, and they were more likely to have short weekend sleep duration. They also had higher average midsleep differences, higher average CSHQ scores, and were more likely to have a CSHQ-indicated sleep problem. *Postnatal depression:* mothers with clinically significant postnatal depressive symptoms (T2) were more likely to perceive a concurrent infant sleep problem. At 3 years (T3), children whose mothers had experienced clinically significant postnatal depressive symptoms at T2 had shorter average sleep durations and were more likely to have short weekend sleep. They also had higher average CSHQ scores and were more likely to have a CSHQ-indicated sleep problem. *Depression at 3 years post-birth:* children of mothers with clinically significant depressive symptoms at T3 were more likely to have short weekend sleep, higher average CSHQ scores, and exhibit perceived and CSHQ-indicated sleep problems.

**Table 2.** Infant and preschooler sleep measures by prenatal, postnatal and 3-year post-birth maternal depressive symptoms above or below clinical cutoffs

12 weeks, % (95% CI)	Prenatal				Postnatal				Child 3 years			
	EPDS ≥ 13		EPDS < 13		EPDS ≥ 13		EPDS < 13		K-10 ≥ 12		K-10 < 12	
	*n*		*n*		*n*		*n*		*n*		*n*	
Night wakings (3+)	16	11.2 (6.8 to 17.1)	62	8.7 (6.8 to 11.0)	11	15.5 (8.5 to 25.2)	67	8.6 (6.8 to 10.7)				
Night LSRSP (≤4 hours)	30	21 (14.9 to 28.2)	80	11.3 (9.1 to 13.8)**	14	19.7 (11.8 to 30.1)	97	12.4 (10.2 to 14.9)				
Day LSRSP (≤1 hour)	15	10.5 (6.3 to 16.3)	67	9.4 (7.5 to 11.8)	9	12.7 (6.5 to 21.9)	73	9.3 (7.4 to 11.5)				
Perceived sleep problem	35	24.5 (18.0 to 32.0)	172	24.2 (21.2 to 27.5)	30	42.3 (31.3 to 53.9)	177	22.6 (19.8 to 25.7)***				
3 Years, % (95% CI) OR mean ± SD												
Sleep duration												
24 hour sleep duration, hours^a^												
Weekday	137	11.06 ± 1.42	693	11.41 ± 1.26**	66	10.97 ± 1.37	764	11.38 ± 1.28*	128	11.16 ± 1.41	705	11.38 ± 1.27
Weekend	134	10.82 ± 1.57	693	11.39 ± 1.23***	63	10.76 ± 1.53	764	11.33 ± 1.27**	125	11.15 ± 1.66	705	11.32 ± 1.23
Short sleep duration week (<10 hours/24 hours)	16	11.7 (7.1 to 17.8)	50	7.2 (5.5 to 9.3)	8	12.1 (5.9 to 21.6)	58	7.6 (5.9 to 9.6)	14	10.9 (6.4 to 17.2)	52	7.4 (5.6 to 9.5)
Short sleep duration weekend (<10 hours/24 hours)	25	18.7 (12.8 to 25.9)	48	6.9 (5.2 to 9.0)***	14	22.2 (13.3 to 33.6)	59	7.7 (6 to 9.8)***	17	13.6 (8.4 to 20.4)	56	7.9 (6.1 to 10.1)*
Social jetlag												
Midsleep difference, mins	128	19.91 ± 23.13	674	15.25 ± 20.58*	61	15.94 ± 18.38	741	16.00 ± 21.31	119	15.85 ± 20.06	686	16.01 ± 21.25
Social jetlag ≥ 1 hour	12	9.4 (5.2 to 15.3)	39	5.8 (4.2 to 7.7)	3	4.9 (1.4 to 12.5)	48	6.5 (4.8 to 8.4)	5	4.2 (1.6 to 9.0)	46	6.7 (5.0 to 8.8)
CSHQ												
CSHQ total	143	44.73 ± 8.89	708	41.67 ± 8.37***	71	45.45 ± 9.45	780	41.91 ± 8.38***	133	45.71 ± 9.41	721	41.54 ± 8.19***
CSHQ-indicated sleep problem^b^	96	67.1 (59.2 to 74.4)	348	49.2 (45.5 to 52.8)***	52	73.2 (62.2 to 82.5)	393	50.4 (46.9 to 53.9)***	94	70.7 (62.6 to 77.9)	352	48.8 (45.2 to 52.5)***
Perceived sleep problem												
Moderate-large problem	29	20.3 (14.3 to 27.4)	114	16.1 (13.6 to 19.0)	14	19.7 (11.8 to 30.1)	130	16.7 (14.2 to 19.5)	35	26.3 (19.4 to 34.3)	110	15.3 (12.8 to 18.1)**

^a^Outlier of 4 hours sleep duration removed.

^b^Defined as total CSHQ Score > 41.

*p < .05, **p < .01, ***p < .001.

### Binary logistic regression associations between maternal depressive symptomology and child sleep

Tables 3 and 4 present the results of binary logistic regression models investigating longitudinal associations between maternal depressive symptoms and infant and preschooler sleep, respectively. All models controlled for key demographic factors; preschooler models also controlled for prior sleep variables. *Infant sleep:* There were no independent associations between prenatal depressive symptoms and infant sleep variables at 12 weeks. However, concurrent postnatal depressive symptoms were independently associated with more night waking (≥ 3 vs. < 3 wakings/night), shorter night LSRSP (≤4 vs. >4 hours), and perceived problem sleep. *Preschooler sleep:* Preschoolers whose mothers had experienced more depressive symptoms during pregnancy were more likely to exhibit short sleep, both during the week (OR 1.084 [1.020-1.152]) and on weekends (OR 1.079 [1.017-1.144]). There were no associations between postnatal depressive symptoms and preschooler sleep variables. However, concurrent maternal depressive symptoms at 3 years post-birth were independently associated with both CSHQ-identified and parent-perceived sleep problems.

**Table 3.** Adjusted^a^ associations between maternal depressive symptoms^b^ and binary infant sleep outcomes

	Odds ratio (95% CI)			
	Night wakings ≥ 3	Night LSRSP ≤ 4 hours	Day LSRSP ≤ 1 hour	Perceived problem
Prenatal EPDS	1.00 (0.95 to 1.06)	1.05 (1.00 to 1.10)	1.02 (0.96 to 1.07)	0.98 (0.94 to 1.02)
12 weeks postnatal EPDS	1.07 (1.01 to 1.13)*	1.05 (1.00 to 1.11)*	1.04 (0.98 to 1.10)	1.13 (1.09 to 1.18)***

NB: Odds ratios indicate the change in odds of a given sleep outcome for each one-point increase in EPDS/K-10 score. The SDs for the EPDS were 4.83 (prenatal) and 4.27 (postnatal).

^a^In addition to depression at both time points, independent variables included in the infant sleep models were maternal ethnicity at T1, maternal age at T1, NZDep level at T1, child age at T2, and child sex.

^b^All depression measures entered as continuous independent variables.

*p < .05, **p < .01, ***p < .001.

**Table 4.** Adjusted^a^ associations between maternal depressive symptoms^b^ and binary preschooler sleep outcomes

	Odds ratio (95% CI)			
	Short week (<10 hours)	Short weekend (<10 hours)	CSHQ-indicated problem^c^	Perceived problem
Prenatal EPDS	1.08 (1.02 to 1.15)*	1.08 (1.02 to 1.14)*	1.03 (0.99 to 1.07)	1.03 (0.98 to 1.08)
12 weeks postnatal EPDS	0.96 (0.89 to 1.03)	1.02 (0.95 to 1.09)	1.04 (1.00 to 1.08)	0.97 (0.92 to 1.02)
3 years Post-birth K-10	1.01 (0.96 to 1.07)	1.02 (0.97 to 1.07)	1.07 (1.03 to 1.10)***	1.07 (1.03 to 1.11)**

NB: Odds ratios indicate the change in odds of a given sleep outcome for each one-point increase in EPDS/K-10 score. The SDs for the EPDS were 4.83 (prenatal) and 4.27 (postnatal). The SD for the K-10 was 5.40.

^a^In addition to depression at all time points, independent variables included in the preschooler sleep models were maternal ethnicity at T1, maternal age at T1, NZDep level at T1, child age at T3, and T2 sleep variables (night waking, night LSRSP, day LSRSP, and perceived sleep problem).

^b^All depression measures entered as continuous independent variables.

^c^Defined as total CSHQ Score > 41.

*p < .05, **p < .01, ***p < .001.

## Discussion

This study contributes to our nascent understanding of the relationships between maternal depression and sleep in young children. Bivariate analyses showed clinically significant prenatal depressive symptoms were associated with short night LSRSP in infants aged 12 weeks, and with shorter sleep duration, greater average midsleep difference, higher average CSHQ score, and higher rates of CSHQ-identified sleep problems in preschoolers aged 3 years. Clinically significant postnatal depressive symptomology was associated with several parallel infant sleep variables, as well as subsequent preschooler sleep outcomes.

After controlling for key maternal demographic variables, infant sleep characteristics, and postnatal and concurrent maternal depressive symptoms, prenatal depressive symptoms continued to be independently associated with short week and weekend sleep duration in preschoolers. Adjusted models showed no prospective associations between prenatal depression and infant sleep. Consistent with established literature [47–50], we found concurrent relationships between maternal depression and infant night sleep characteristics, and clinically significant preschooler sleep problems, indicated by the CSHQ. Perhaps reflecting these associations, we also found that mothers with greater depressive symptomology were more likely to experience their child’s sleep as problematic at both time points.

The marginal effect sizes indicated by the odds ratios in the logistic regression models belie meaningful increases in odds of short preschooler sleep duration conferred by maternal depression. For example, the odds ratios for short sleep indicate that for every one-point increase on the prenatal EPDS, the odds of a child exhibiting shorter-than-recommended sleep at 3 years increase by 8.4% and 7.9%, for week and weekend sleep, respectively. An increase of one standard deviation on the prenatal EPDS (4.83 points) therefore equates to a 40.6% (week) and 38.2% (weekend) greater odds of short sleep. This is particularly striking when we note that this relationship exists over and above how the children slept as infants, as well as mothers’ postnatal and concurrent depressive symptoms, and key demographic variables including ethnicity and SEP.

While this study contributes to an emerging pattern within the literature that shows prenatal depression is associated with subsequent child sleep, broadly defined, it also adds to the inconsistencies. In line with some findings [18] and contrasting with others [17, 51], our adjusted models showed no associations between prenatal depression and infant sleep. Together, findings to date suggest clear relationships are difficult to detect at such an early stage of infant development when sleep is highly variable [52]. Our findings also contribute to inconsistency regarding which specific aspects of preschooler sleep are affected by prenatal depression [17, 21, 53]. For example, after controlling for covariates O’Connor et al. [21] found an association between prenatal maternal mood and preschooler sleep problems but not sleep duration, whereas we found the reverse: In our adjusted models, prenatal mood was associated with week and weekend short sleep durations, but not sleep problems. Like us, Toffol et al. [20] found an association between prenatal mood and sleep duration, but they also found an association with sleep problems where we did not. Methodological factors may account for the variability in these associations, such as sample demographics and measures used. For example, Toffol et al. [20] used The Sleep Disturbance Scale for Children (SDSC) with their sample of Finnish participants. The SDSC differs from the CSHQ in terms of sleep problems indexed and populations with whom the measures were validated [39, 54]. As more evidence accumulates on this important topic, meta-analyses should confirm the specific dimensions of pediatric sleep health most impacted. Inconsistences could also stem from the time at which depressive symptoms were measured during pregnancy. Though several studies suggest prenatal depression is highly stable across pregnancy [55, 56], others show heterogeneity in symptom trajectories [57]. Future research could explore potential sensitive periods in gestation that are especially vulnerable to the effects of prenatal depression on child sleep, based on the developmental timing of relevant brain regions [58]. The question of sensitive periods was considered by Toffol et al. [20], but the stability of depressive symptoms in their sample precluded a thorough examination of pregnancy stage-specific associations.

Our findings underscore the public health message sent by Toffol et al. [20] that treatment interventions designed to support maternal mental health during pregnancy have the potential to alleviate at least some of the sleep problems that affect a large pediatric population. Compared to postnatal depression, prenatal depression is an under-recognized risk factor for suboptimal family health outcomes [59]. Prior research indicates that help-seeking rates among women experiencing depression during pregnancy are low [60]. This highlights the need for effective population-level policies and intervention programs, including robust systems to identify those who are suffering. Depression screens should be used routinely in preconception and prenatal care. Those at risk should be supported with mental health services that are fit for purpose across and beyond the perinatal period [61]. The current findings suggest early intervention could help prevent shorter-than-recommended sleep in early childhood and therefore support long-term cognitive, emotional, behavioral, and academic functioning in children [6]. Furthermore, effective intervention before and during pregnancy could prevent the continuation or development of maternal depression at later stages [62, 63], and the concurrent child sleep problems associated. Given the complex interplay posited in the transactional model of sleep development [7], improvements in child sleep are likely to also promote maternal sleep and mental health and thus contribute to an “upward spiral” in overall family wellbeing.

To our knowledge, this is the first study to explore the relationships between prenatal depression and infant and preschooler sleep in a large, ethnically and socio-economically diverse sample. It is imperative that research includes Indigenous and other non-white groups. These populations are subject to institutional racism which produces and maintains systemic inequities in the social determinants of health broadly, and sleep health specifically [64–66]. Participation in research is critical to addressing these inequities and upholding rights to health that are clearly outlined in the United Nations Declaration of the Rights of Indigenous Peoples (UNDRIP), as well as in Te Tiriti o Waitangi/The Treaty of Waitangi in NZ [22, 66–68]. Other strengths of the current study include the range of pediatric sleep variables explored, and the incorporation of numerous covariates, including infant sleep characteristics and maternal depressive symptoms measured at earlier and parallel time points.

Our findings should be considered in light of the study’s limitations. First, we used mother-rated measures of child sleep- an established methodology for large-scale studies. We assessed commonly explored variables and included the well-validated CSHQ, but our results cannot be generalized to objectively measured sleep characteristics. While we were able to control for numerous important covariates, we were insufficiently powered to account for all variables known to be relevant to maternal depression and child sleep. For example, research within *Moe Kura* has demonstrated the relevance of factors such as stressful life events and social support [46, 60, 69], neither of which could be considered here. However, prior cross-sectional analyses suggest maternal depression–child sleep relationships are robust to these and other covariates [36]. Despite the strength of our sample’s ethnic and socioeconomic diversity, sample characteristics differed somewhat from those of *Moe Kura’s* baseline cohort [60]. Importantly though, attrition similarly affected our Māori and non-Māori groups, such that neither group differed from their baseline counterparts in terms of prenatal depression prevalence, a key construct of interest. Issues with shared-method variance are possible given that mothers reported on both depressive symptoms and child sleep. Cognitive biases typical of depression might cause mothers to experience (and rate) their children’s sleep as more problematic [48, 70]. Additionally, maternal sleep disturbances—a characteristic of depression—might make mothers more sensitive to their children’s sleep disturbances (e.g. brief wakings), leading to overreporting of child sleep problems [71]. These phenomena could account for the strong relationship between maternal depressive symptoms and sleep problems measured in parallel at both 12 weeks and 3 years post-birth. However, they are unlikely to account for our longitudinal findings, as prenatal depressive symptoms showed effects on preschooler sleep duration that were independent of concurrent maternal depressive symptoms.

## Conclusion

Our findings suggest short sleep is one pathway by which maternal depression confers risk for suboptimal child health outcomes. Clinically significant depressive symptomology in the third trimester of pregnancy was associated with shorter average weekday and weekend sleep duration among children at preschool age. Prenatal depressive symptoms were associated with shorter-than-recommended sleep at age 3 years, over and above the impact of key maternal demographic variables, prior infant sleep characteristics, and postnatal and parallel maternal depressive symptoms. Clinically significant postnatal depressive symptoms were also associated with several concurrent and future child sleep characteristics in bivariate models, but only the concurrent relationships between postnatal depression and child sleep remained after adjusting for covariates. Based on these findings, we echo calls for earlier and better mental health support for mothers. Support should be fit for purpose across and beyond the perinatal period. Appropriately addressing maternal depression is likely to benefit child sleep and the myriad domains of child and family well-being that are supported by pediatric sleep health.

## Data Availability

The data used in this research can be shared on reasonable request to the corresponding author and with agreement from the original *Moe Kura* research team.
